# Microalgae for the production of lipid and carotenoids: a review with focus on stress regulation and adaptation

**DOI:** 10.1186/s13068-018-1275-9

**Published:** 2018-10-04

**Authors:** Xiao-Man Sun, Lu-Jing Ren, Quan-Yu Zhao, Xiao-Jun Ji, He Huang

**Affiliations:** 1 College of Biotechnology and Pharmaceutical Engineering, Nanjing Tech University, No. 30 South Puzhu Road, Nanjing, 211816 People’s Republic of China; 2 School of Pharmaceutical Sciences, Nanjing Tech University, No. 30 South Puzhu Road, Nanjing, 211816 People’s Republic of China; 3 State Key Laboratory of Materials-Oriented Chemical Engineering, Nanjing Tech University, No. 5 Xinmofan Road, Nanjing, 210009 People’s Republic of China; 4 Jiangsu National Synergetic Innovation Center for Advanced Materials (SICAM), Nanjing, People’s Republic of China

**Keywords:** Microalgae, Stress, Lipid, Carotenoids, Growth-promoting agents, Adaptive laboratory evolution

## Abstract

Microalgae have drawn great attention as promising sustainable source of lipids and carotenoids. Their lipid and carotenoids accumulation machinery can be trigged by the stress conditions such as nutrient limitation or exposure to the damaging physical factors. However, stressful conditions often adversely affect microalgal growth and cause oxidative damage to the cells, which can eventually reduce the yield of the desired products. To overcome these limitations, two-stage cultivation strategies and supplementation of growth-promoting agents have traditionally been utilized, but developing new highly adapted strains is theoretically the simplest strategy. In addition to genetic engineering, adaptive laboratory evolution (ALE) is frequently used to develop beneficial phenotypes in industrial microorganisms during long-term selection under specific stress conditions. In recent years, many studies have gradually introduced ALE as a powerful tool to improve the biological properties of microalgae, especially for improving the production of lipid and carotenoids. In this review, strategies for the manipulation of stress in microalgal lipids and carotenoids production are summarized and discussed. Furthermore, this review summarizes the overall state of ALE technology, including available selection pressures, methods, and their applications in microalgae for the improved production of lipids and carotenoids.

## Background

With the increase of world population and energy demand, the search for renewable energy resources has become a critical issue. Microalgae have been recognized as a potential source of livestock feed, pharmaceuticals, and alternative fuels [1, 2]. Microalgae can utilize one or more of their three major metabolic modes, photoautotrophy, heterotrophy, and mixotrophy, depending on light conditions and carbon availability [3, 4]. In any of these modes, microalgae can provide an abundance of value-added products, and more recently, interest has focused on lipids and carotenoids. The lipid content of microalgae is usually in the range of 20–50% of the cell dry weight, and can be as high as 80% under certain conditions [5]. Microalgal lipids are classified into two groups according to their carbon number. Fatty acids having 14–20 carbons were used for the production of biodiesel, and polyunsaturated fatty acids (PUFAs) with more than 20 carbon atoms were used as health food supplements especially docosahexaenoic acid (DHA) and eicosapentaenoic acid (EPA). In addition, microalgae are also known to produce carotenoids, which are responsible for light harvesting in photosynthetic metabolism. The absorbed energy can be transferred to chlorophylls by primary carotenoid like lutein, thus expanding the light-absorbing spectrum of microalgae [6]. Moreover, due to their anti-oxidant properties, carotenoids play an important role in alleviating certain cancers, premature aging, cardiovascular diseases, and arthritis [7, 8]. Furthermore, carotenoids are also often used as coloring agents and dyes because of their intrinsic color [9, 10]. Among the well-known microalgal carotenoids, β-carotene, astaxanthin and lutein have the highest current market potential.

Microalgae can overproduce lipids or carotenoids under stress condition such as high salt, high light, or nutrient limitation [11, 12]. For instance, lipid accumulation in *Dunaliella* sp. and *Chlorella vulgaris* was significantly increased under high-salinity stress, reaching 70% and 21.1%, respectively [13, 14]. Similarly, when salt concentration was increased from 4 to 9%, β-carotene yield of *Dunaliella salina* was increased by 30-fold [15]. However, stress-based strategies usually influence cell growth in an adverse way and also decrease the yield of desired products. Furthermore, the formation of reactive oxygen species (ROS) serves as an important component of the cellular responses to the stress condition. Consequently, an increase of biomass by two-stage processes and reduction of oxidative stress using phytohormones or antioxidants were developed to mitigate the negative aspects of stress-based strategies [16, 17]. Since such two-stage processes require complex control strategies, at least in theory, it would be easier to develop new production strains that are genetically capable of optimal growth under the inducing stress conditions. Accordingly, transcription factor engineering was actively developed to improve the production of lipids or carotenoids [18–20]. Moreover, a guideline was proposed for stress-driven adaptive evolution experiments. ALE has been widely utilized in bacteria and fungi to enhance their metabolic phenotypes or their tolerance to particular stress conditions [21, 22]. By the same token, the ALE studies enjoy many advantages offered by the microalgae cells: (1) the majority of the microalgae have simple nutrient requirement; (2) it is easy to cultivate them in the laboratory and (3) it take shorter time for the microalgae cells to grow and their cultivation can span several generations in several months or weeks. In addition, compared to the random mutagenesis methods, ALE having sequential serial passages serves as a comparatively easy approach for the identification of the major mutations relevant with the improvement due to its low mutation frequency. Thus, it has recently been proven as an innovative and effective tool to improve the strain properties of the microalgae, and this field still is certainly open to innovations in the future.

The existing literature mainly focused on emphasizing the effect of various stresses on the production of lipids or carotenoids, or discussed the advantages of metabolic engineering for the improvement of microalgae strain [23–25]. However, in this review, we summarize recent works on manipulation of stress factors, including cultivation models and the development of novel stress-tolerant microalgae strains, which is mainly focused on overcoming the negative effects of stress-based strategies. More interestingly, we review the basics of ALE, including selection pressures and methods, and based on this, we summarize the practicability of different ALE approaches for optimizing the production of lipids and carotenoids in microalgae. In addition, the challenges and future perspective of stress regulation and -adaptation strategies were summarized.

## Manipulation of stresses by different cultivation modes

**Fig. 1:**
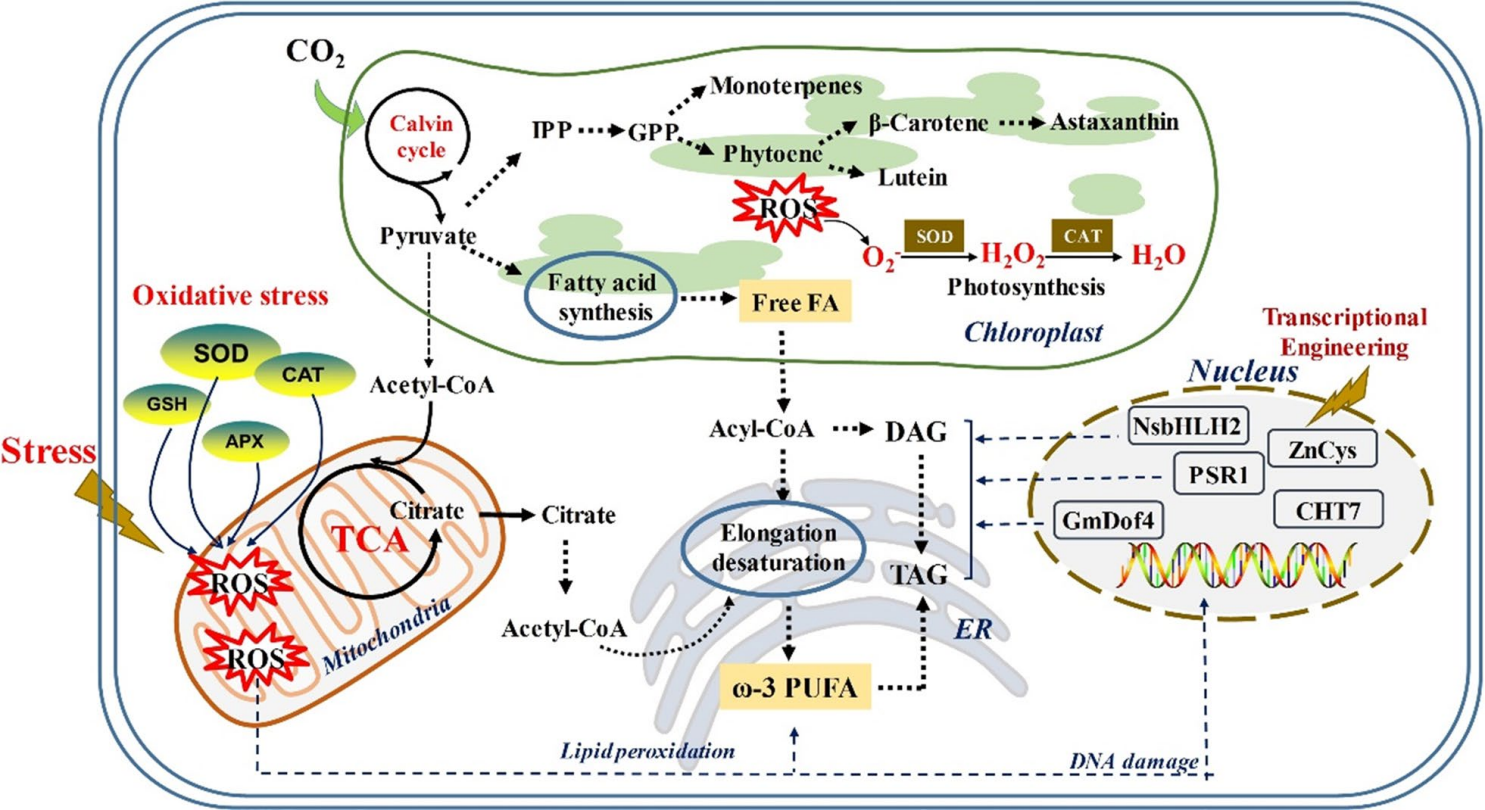
Oxidative damage under stress conditions and manipulation of stresses by transcriptional engineering. *GSH* glutathione, *ER* endoplasmic reticulum

Lower biomass and suffering oxidative injury serve as the two major consequences of cellular responses to the stress-based strategies. It has been well established that ROS can react instantaneously and nonspecifically with essential biological molecules, resulting in the alteration of cellular functions by leading to lipid peroxidation, protein oxidation, and DNA damage (Fig. 1). Therefore, increasing the biomass and maintaining ROS detoxification under stress conditions is paramount for the economically viable production of lipids or pigments.

### Two-stage cultivation strategies

#### Abiotic stresses

**Fig. 2:**
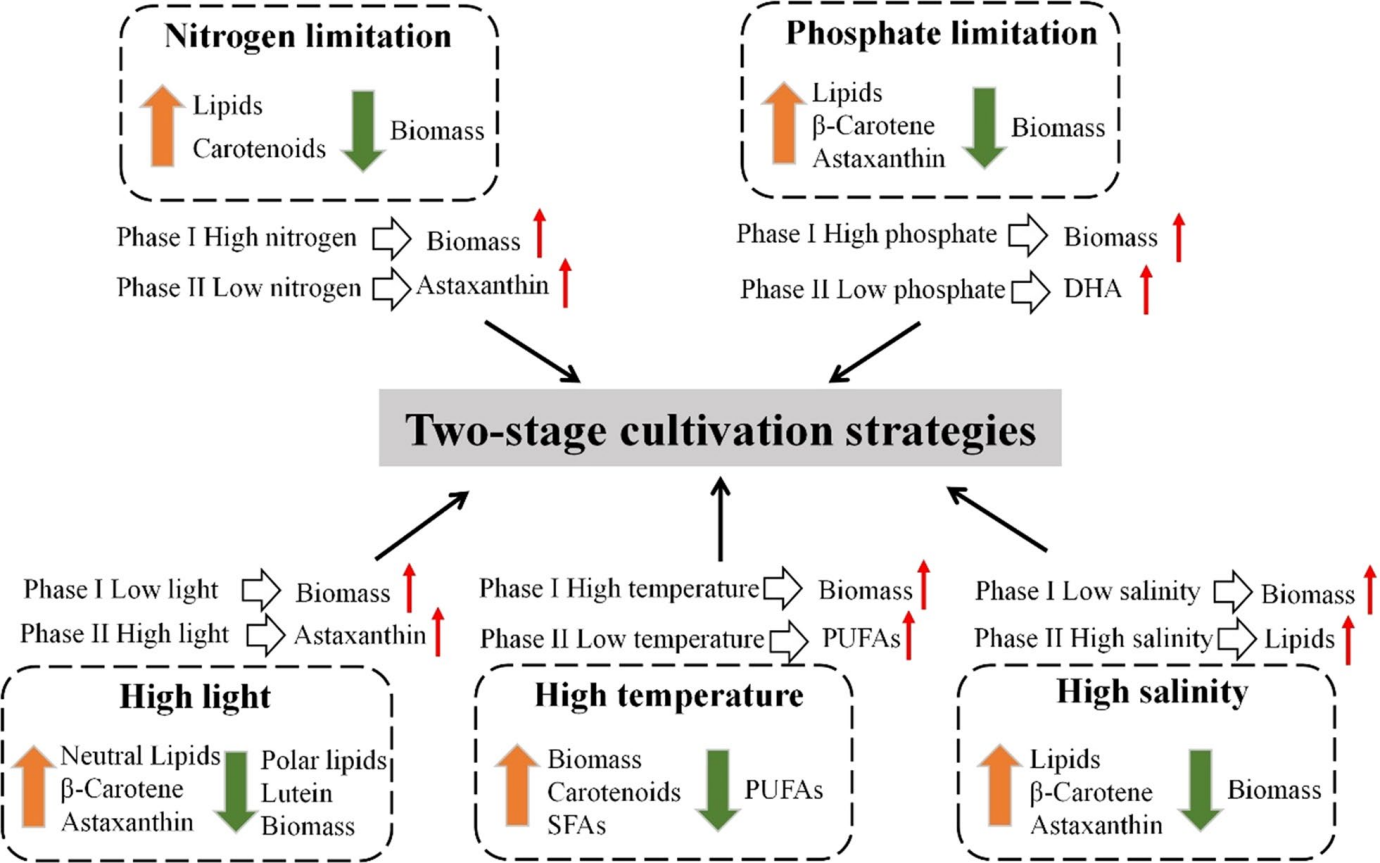
Effects of typical nutrient- and environmental stresses on the production of lipid and carotenoids in microalgae, and the resulting two-stage cultivation strategies used to overcome the biomass limitation imposed by the stress conditions

To resolve the conflict between cell growth and the production of valuable molecules, a general countermeasure is two-stage cultivation strategy, dedicating the first stage with optimum growth conditions to gain the maximum biomass production, while reserving the second process for the accumulation of lipids or carotenoids under various stress conditions (Fig. 2). In general, lipids can be over-produced by microalgae by introducing stress at the second cultivation stage, for instance nitrogen depletion [26], light intensity [27], temperature [28], salt concentration [29], or iron concentration in the second stage [30]. In one approach, microalgae were grown under red LEDs (660 nm) in the first phase to obtain the maximum biomass production, and stressed in the second phase using green LEDs (520 nm) to stimulate lipid accumulation [31]. Similarly, a two-stage culture strategy was implemented to increase the biomass of *Isochrysis galbana* under sufficient nutrients, followed by cultivation under low-salt stress, which increased the lipid content from 24 to 47% [32]. In *Nannochloropsis oculata*, the lipid yield obtained in a two-stage process was 2.82-times over that being generated through the conventional single-stage batch cultivation [33].

Compared to lipids, the algae-based production of pigments is well established at a large scale. Two-stage processes can be more readily scaled up and are amenable to outdoor production [30]. High-light stress is the best induction technique for the overproduction of carotenoids in a number of species. Increasing the light intensity resulted in an enhanced lutein productivity of 3.6 mg L^−1^ day^−1^ in *Desmodesmus* sp., as well as a maximum β-carotene production of 30 pg cell^−1^ day^−1^ in *D. salina* [34, 35]. The two-stage strategy with light stress has been successfully performed for carotenoids production, but it is most commonly applied in the production of astaxanthin. A richer astaxanthin product (4% of dry biomass) was yielded through a two-stage cultivation system in conjunction with light stress, among which a final astaxanthin productivity of 11.5 mg L^−1^ day^−1^ was obtained [36]. Such sequential manufacturing system is also applied for the manufacturing of the astaxanthin through the microalga *Haematococcus pluvialis* [37, 38]. Similarly, a two-stage process with a switch in light intensity was also applied to enhance the lutein productivity of *Scenedesmus obliquus* [39]. In a recent study, an innovative staged cultivation method was proposed to overcome the limiting factors associated with the growth of *H. pluvialis*, which resulted in a 1.16-fold increase in biomass concentration [40]. Moreover, temperature also has strong effect on carotenoids production by influencing the enzymes involved in carotenoids biosynthesis. It has been reported that high temperatures led to an increase of lutein accumulation in *Muriellopsis* sp. and *Scenedesmus almeriensis*. The effects of day- and night-time temperature on *H. pluvialis* were studied, and the results indicated that raising the day- or night-time temperature could stimulate accumulation of astaxanthin during the night up to a temperature of 28 °C [41]. However, insufficient work has been done on carotenoids production using two-stage process combined with high-temperature stress.

#### Autotrophy/heterotrophy regimes

Interestingly, a novel sequential heterotrophy–dilution–photoinduction process was developed to prevent the reduction of biomass. In this approach, microalgae are grown heterotrophically first to obtain a high cell density, after which the culture medium is diluted to a suitable concentration. Subsequently, it was transferred to a brightly lit environment for photoinduction. A novel approach called “sequential heterotrophy–dilution–photoinduction” was developed, in which the lipid content of *Chlorella vulgaris, Chlorella pyrenoidosa* and *Chlorella ellipsoidea* was increased by 84.57%, 70.65%, and 121.59%, respectively [42]. Similarly, a maximum lipid content of 50.5% (of dry weight) was achieved using heterotrophy–photoinduction cultivation regime, representing a 69.3% increase over that of single heterotrophic cultivation [43]. More importantly, proteomic analysis revealed that ATP synthase and acyl-CoA dehydrogenase were down-regulated, while glucose-1-phosphate adenylyltransferase and malate dehydrogenase were markedly up-regulated [43]. Furthermore, this system has been utilized for biomass and astaxanthin production in *H. pluvialis* [44]. Recently, using a two-stage heterotrophy/photoinduction culture strategy, the lutein productivity of *Scenedesmus incrassatulus* was improved by 60% in comparison with the autotrophic fed-batch culture [45]. The productivity of total metabolites and lipids can be increased using these strategies in the same biomass obtained at the end of the growth cycle. However, such two-stage cultivation strategies can consume more energy than comparable one-stage systems, especially if harvesting the biomass is essential for entry into the second fermentation stage [46]. Moreover, it is difficult to determine the timepoint of fermentation exactly, which results in the instability of the production.

### Supplementation of growth-promoting agents

#### Regulating biosynthetic pathways

In addition to two-stage cultivation strategies, phytohormones are also able to induce the production of lipid or pigments in microalgae by adjusting the internal biochemical pathways [16], which serves as growth-promoting agents within the single-stage cultivation process (Table 1). Auxins play an important role in the growth of plants and microalgae by regulating cell division and expansion [47]. In *S. obliquus*, the biomass yield was increased by 1.9–2.5-folds through addition of 10^−5^ M Indole-3-acetic acid (IAA) and diethyl aminoethyl hexanoate (DAH), respectively, whereby the lipid content of microalgae grew to 100 mg/g-DCW due to the impact of both phytohormones [48]. Moreover, improved growth and lipid accumulation under the impact of the IAA and corresponding analogs has also been reported in *C. vulgaris* [49], *C. pyrenoidosa* and *Scenedesmus quadricauda* [50].

**Table 1:** Manipulation of stress factors by phytohormones

Species	Phytohormones	Stress	Performance	References
*Scenedesmus quadricauda*	ABA	Nitrogen-deficient stress	The dry biomass yield was increased up to 2.1 fold	[56]
*Chlorella sorokiniana*	IAA, DA-6	Nitrogen-limited stress	Growth and lipid accumulation were both promoted and phytohormones enhances CAT and SOD enzyme activities	[52]
*Nannochloropsis oceanica*	ABA, CKs	Nitrogen-depletion stress	Exogenous CKs stimulate cell-cycle progression, but ABA acts as both an algal growth repressor and a positive regulator in response to stresses	[70]
*Chlamydomonas reinhardtii*	IAA, GA_3_, KIN, TRIA, ABA	Nitrogen-limited stress	All five of the tested phytohormones significantly increased microalgal growth, particularly in nitrogen-limited media	[54]
*Chlamydomonas reinhardtii*	ABA	Osmotic and salt stresses	ABA treatment markedly reduced ROS generation and enhanced gene expression of the antioxidant enzymes	[58]
*Chlorella vulgaris*	IAA, PAA, IBA, NAA	Oxidative stress	All auxins can suppress lipid peroxidation and hydrogen peroxide accumulation	[49]
*Chlamydomonas reinhardtii*	ABA	Oxidative stress	Addition of ABA improve the growth of this alga	[67]

Moreover, auxin treatments combined with stress conditions can further stimulate cell growth and the lipid production of microalgae [51]. Recently, under stress induced by a 50% nitrogen limitation, the highest biomass concentration and lipid productivity were achieved by treating *Chlorella sorokiniana* with IAA [52]. Furthermore, the up-regulated expression of *rbcL* (ribulose 1, 5-bisphosphate carboxylase/oxygenase) and *accD* (acetyl-CoA carboxylase) genes suggested that IAA can regulate the certain metabolic pathways related to carbon fixation and lipid biosynthesis [50]. In another study, Liu et al. [53] founded that 1-naphthaleneacetic acid (NAA) exhibited a more pronounced promoting effect on cell growth and lipid productivity of *C. vulgaris* than abscisic acid (ABA) and 2,4-dichlorophenoxyacetic acid (2,4-D), and further analysis showed that NAA treatment can up-regulate the *KAS1* and *SAD* genes expression which regulated fatty acids biosynthesis in microalgae. It has been reported that the stimulated effect of IAA on microalgae growth was more obvious under nitrogen limitation condition [54]. A novel stepwise strategy was developed to improve both growth and lipid production in *Dunaliella tertiolecta*, in which 2, 4-D addition at the first phase improved biomass productivity by 40%, and then salt stress was applied to increase lipid content from 24 to 70% [55]. ABA can enlarge cell diameter and promote cell division to alleviate growth limitation caused by stress condition, although it forces microalgae cell to enter the resting phase earlier. For example, exogenous addition of abscisic acid (ABA) increased the biomass and SFA content of *S. quadricauda* up to 2.1-fold and 11.17% compared to nitrogen-deficient cells, respectively [56], whereby exogenous ABA also improved the cells’ tolerance to higher salinity and osmotic stress [57, 58].

Similarity, phytohormones also can enhance carotenoids production by working as metabolism enhancers. After treatment with methyl jasmonate and gibberellin A3, *H. pluvialis* cells accumulated more astaxanthin than the control, which might be due to the up-regulation of β-carotene ketolase genes [59]. Gao et al. [60, 61] reported that the astaxanthin productivity of *H. pluvialis* can be increased by supplementing 25 or 50 mg/L jasmonic acid (JA) and salicylic acid (SA), and further analysis found that the promoting mechanisms were dose dependent. For instance, 25 mg/L JA treatment exhibited greater effect on the expression of *pds*, *crt*R-B and *lyc* genes, while 50 mg/L JA impacted the expression of *ipi*-*1*, *ipi*-*2*, *psy*, *crt*R-B and *crt*O genes more significantly. In addition, under 5 mg/L fulvic acid induction, the astaxanthin content of *H. pluvialis* was increased by 86.89%, in which the transcription levels of phytoene desaturase (PDS), lycopene cyclase gene (LCY) and β-carotene hydroxylase (CHY) genes was increased by 69.3-, 1.2-, and 18.1-fold, respectively [62]. In conclusion, addition of phytohormones strategy is a sustainable and economical strategy, and it can easily be applied for large-scale production of lipids or carotenoids.

#### Alleviating oxidative stress

It is worth mentioning that increasing experimental evidence links phytohormones to a reduction of oxidative stress. ROS are unavoidable by-products of many metabolic pathways that are active under stress conditions [63]. The cellular ROS of the highest importance include the hydrogen peroxide (H_2_O_2_), superoxide anion (O_2_^−^), peroxyl radicals (LOO^−^), lipid hydroperoxides (LOOH), and hydroxyl radical (^−^OH) [64]. To scavenge ROS, microalgae have evolved enzymatic (*SOD* superoxide dismutase, *CAT* catalase, *APX* ascorbate peroxidase) as well as non-enzymatic (ascorbate, glutathione) antioxidant defense mechanisms (Fig. 1). Nevertheless, when cells are exposed to stress, balance between the ROS production and elimination is disturbed. This results in the damage via the oxidation of cellular components, i.e., “oxidative stress” [65].

Increasing evidences suggested that phytohormones can regulate the oxidative stress response of microalgae [66 换一个新的]. For example, the activities of CAT and APX were markedly increased in response to oxidative stress in ABA-treated *Chlamydomonas reinhardtii* [67]. Likewise, addition of ABA can enhance the gene expression of the APX and CAT for mitigating oxidative stress generated by osmotic and salt stresses [58]. Chokshi et al. [68] and Wu et al. [69] showed that the phytohormone content was significantly higher in nitrogen-depleted microalgae cells than in nitrogen-replete condition, likely because intracellular oxidative species and phytohormones interact with each other to fine-tune the oxidative stress response. In the oleaginous microalga *Nannochloropsis oceanica*, Lu et al. [70] also found that the key genes involved in the ABA biosynthesis pathway were up-regulated in nitrogen-starved cells, indicating the function of the ABA on the release of the stress damage. Moreover, phytohormones are often associated with the signaling induced by ROS. For instance, the H_2_O_2_ and auxin create the antagonistic impacts over the gene activation and cell cycle [71]. H_2_O_2_ treatment can suppress the expression of auxin-responsive genes via mitogen-activated protein kinase activation [72]. Recently, Khasin et al. [73] suggested that ABA can participate in ROS signaling pathways and alter ROS production and scavenging, which is a potential new mechanism for the regulation of oxidative stress.

In fact, antioxidants have been used more extensively than phytohormones to promote the scavenging of intracellular ROS in microalgae. But almost, all of such studies were focused on PUFAs production, which might be for the reason that the unsaturated double bonds in PUFAs are chemically more prone to oxidation. For instance, DHA productivity of *Crypthecodinium cohnii* was increased from 20 to 44% by adding the sesamol [74]. Additionally, Gaffney et al. [75] enhanced the antioxidant ability of the microalgae *Schizochytrium* sp. through the facilitations of the flaxseed oil. Moreover, with the treatment of ascorbic acid, DHA yield of *Schizochytrium* sp. was increased from 26.5 to 38.3 g/L, which accompanied with the lower ROS levels and higher antioxidant capacity [17]. Supplementing butylated hydroxytoluene or propyl into the media led to the increase of lipid and biomass levels in the thraustochytrids, with propyl gallate being the more effective of the two antioxidants [76]. Therefore, the combination of antioxidants and stress-based strategies appears to be a practical cultivation mode to improve the growth of microalgae.

#### Co-cultivation of microalgae with the growth-promoting bacterium

For the reduction of the production cost and improvement of lipid productivity, microalgae should be simultaneously cultivated in low-cost cultivation systems, such as co-cultivations [77]. In the mixed culture, microalgae could act as an oxygen generator for the bacteria while the bacteria provided CO_2_ to microalgae [77]. Moreover, certain class of bacteria can stimulate microalgae growth by producing growth-promoting factors, including trace metals, vitamins, phytohormones, and chelators [77]. Do Nascimento et al. [78] showed that inoculation with the bacterium *Rhizobium* strain resulted in increments of up to 30% in lipids accumulation of the oleaginous microalgae *Ankistrodesmus* sp., and this stimulation effect was apparently related to indol 3-acetic acid and/or vitamin B_12_ produced by the bacterium. Many studies reported that the growth of *C. vulgaris* was significantly enhanced in the presence of *Azospirillum brasilense* that a bacterium can produce the plant hormone indole-3-acetic acid [79, 80]. When microalgae *Chlorella* sp. was co-cultured with the growth-promoting bacterium *Azospirillum* sp., the lipid content was greatly increased from 6.76% under monoculture to 32.94% under co-culture system [81]. However, although microalgae–bacteria co-cultivation has been comprehensively described, the positive effects of bacterial on lipid production are mostly speculative. In the future, it is needed to select and characterize more growth-promoting bacteria for improving microalgae cultivation.

## Countering the negative effects of stress via transcription factor engineering

Transcription factors (TFs) are global regulators of biological pathways that act by up- or downregulating target genes, which have been widely used to construct robust microalgal strains (Table 2). Compared to directed modification of specific enzymes, TFs engineering can provide more substantial metabolic modification by controlling multiple steps in a pathway [82]. In fact, diverse “omics” technologies, including genomics, transcriptomics, lipidomics, proteomics, and metabolomics, are powerful tools to identify stress-response genetic targets under stress condition [83–85]. For example, using a combined omics (transcriptomic, proteomic and metabolomic) analysis, about 70 TFs genes were identified in *C. reinhardtii* that are involved in controlling nitrogen-deprivation process [86]. In recent years, an increasing number of TFs function have been confirmed by direct genetic experiments (Fig. 1).

**Table 2:** Transcription factors involved in stresses

Species	Transcription factors	Stresses	Performances	References
*Chlamydomonas reinhardtii*	PHR1	Phosphate starvation	PHR1 acts downstream in the phosphate starvation signaling pathway via binding the promoter of phosphate starvation responsive structural genes	[83]
*Chlamydomonas reinhardtii*	LCR1	CO_2_-limiting stress	LCR1 transmits the low CO_2_ signal to at least three CO_2_-responsive genes and then fully induces carbon-concentrating mechanism	[94]
*Dunaliella bardawil*	WRKY	Salt stress	All the carotenogenic genes can be recognized by WRKY transcription factors	[95]
*Chlamydomonas*	NRR1	Nitrogen starvation	NRR1, a putative SQUAMOSA promoter binding protein-type transcription factor, was proved to be a regulator of N-induced TAG biosynthesis	[86]
*Chlamydomonas reinhardtii*	PSR1	Nitrogen starvation	PSR1 is a pivotal switch that triggers cytosolic lipid accumulation	[93]
*Chlamydomonas reinhardtii*	PSR1	Phosphorus starvation	PSR1 gene is an important determinant of lipid and starch accumulation in response to phosphorus starvation but not nitrogen starvation	[92]
*Chlorella ellipsoidea*	GmDof4	Nitrogen starvation	Increase of lipid content without growth limitation	[90]
*Nannochloropsis salina*	NsbHLH2	Nitrogen limitation	Biomass and FAME productivity was increased by 36% and 33%, respectively	[99]
*Nannochloropsis gaditana*	ZnCys	Nitrogen starvation	Lipid is doubled by attenuation of ZnCys expression	[100]

Dof-type transcription factor was suggested to regulate the transcription of many genes involved in lipid biosynthesis via directly interacting with DNA in their promoter regions. Overexpression of a Dof-type TF (GmDof4) greatly increased lipid production of *Arabidopsis*, and authors found that the acetyl-coenzyme A carboxylase (ACCase) gene was up-regulated in transgenic plants [87]. ACCase is responsible for transforming acetyl-CoA into malonyl-CoA, which is the first and rate-limiting step for the biosynthesis of fatty acids. When overexpressed a Dof-type TF, the total lipid production of *C. reinhardtii* CC-137 was also increased by twofold [88]. In transgenic strain, the expression of the enoyl-ACP-reductase (ENR1) and the sulfolipid synthase (SQD2) genes was greatly up-regulated, which is a key gene related to fatty acid synthase (FAS) and the glycerolipid biosynthesis, respectively [88]. Furthermore, Salas-Montantes et al. [89] investigated the feasibility for improving lipid accumulation of *C. reinhardtii* under nutrient deficiency stress by overexpression of a Dof-type TF (Dof11), and results showed that the lipid content and the proportion of specific fatty acids were all increased. Similarly, a combination of the overexpression of GmDof4 and mixotrophic culture condition was investigated in microalgae *Chlorella ellipsoidea*, and results showed that the lipid content was increased from 46.4 to 52.9% with no impact over the growth rate [90]. Furthermore, analysis results indicated that the expression of 754 genes was up-regulated and that of 322 genes was down-regulated in transgenic *C. ellipsoidea*, which suggested that GmDof4 may regulate genes related to fatty acid, lipid, carbohydrate metabolism, and protein [90].

A MYB-type transcription factors, Pi Starvation Response1 (PSR1), was found to regulate phosphate starvation signaling by up-regulating phosphatases and Pi transporters. Under P starvation, PSR1 mutation of *C. reinhardtii* not only inhibited lipid biosynthesis but also abolished starch production induced by starvation [91]. Furthermore, Bajhaiya et al. [92] examined the metabolic regulatory role of PSR1 under P starvation, and found that its overexpression increased starch biosynthesis of *C. reinhardtii*, which correlated with a higher expression of specific starch metabolism genes such as starch synthase (SSS1) and phosphorylases (SP1). In author study, PSR1 has also been identified as a regulator of TAG biosynthesis in response to nitrogen starvation in *C. reinhardtii*, and its overexpression increased TAG accumulation without inhibiting growth [93]. However, that study did not examine the expression level of downstream target genes and the potential mechanism of PSR1 under P starvation. Moreover, it was reported that MYB-type TF also play a role in CO_2_-responsive genes in *C. reinhardtii* and salt tolerance in *Dunaliella bardawil*, but lack confirmation by direct genetic experiments [94, 95].

A significant landmark was the identification of nitrogen response regulator NRR1 in *Chlamydomonas*. Insertional mutant NRR1 resulted in a reduction of TAG accumulation by 50%, and its expression was unaffected by other nutrient deficiencies, indicating that this regulator was exclusive to nitrogen-deprivation condition [96]. However, the role of NRR1 in other oleaginous microalgae has not been recapitulated. An intriguing alternative approach to overcome growth limitation under nutrient starvation involves controlling cell quiescence. CHT7 is a transcription factor that regulates quiescence and proliferation under nutrient-starved and -replete conditions, and *C. reinhardtii* mutations of CHT7 can promote starvation-induced TAG accumulation without limiting the biomass yield [97]. Another study identified the regulator TAR1 that has pleiotropic function in response to nitrogen deficiency by regulating cell growth and photosynthesis repression [98]. Compared with the wild type, the TAR1-defective *C. reinhardtii* mutant exhibited more pronounced arrest of cell division, resulting in a 10% higher TAG yield [98]. In *Nannochloropsis salina*, overexpression of the transcription factor NsbHLH2 increased biomass productivity by 36% and FAME productivity by 33% under nitrogen-limitation stress [99]. As for knockout of TF, the lipid accumulation regulator ZnCys in *Nannochloropsis gaditana* was knockout by Cas9-mediated insertional attenuation, and results showed that the lipid productivity was doubled without a concomitant growth limitation under nutrient-replete conditions [100]. However, the availability of these TFs needs to be further proven in more oleaginous microalgae.

Additionally, there is a chance of manipulating the stress-responsive promoters for the increased lipid accumulation. To improve lipid biosynthesis under P deprivation in *C. reinhardtii*, Iwai et al. [101] established an overexpression depending on P deprivation construct of a *Chlamydomonas* type-2 diacylglycerol acyl-CoA acyltransferase (DGTT4), where a P starvation-inducible promoter was introduced. Furthermore, the introduction of this heterologous promoter into *Nannochloropsis* sp. also enhanced the lipid accumulation under P starvation [102]. Taken together, transcription factor engineering is a promising methodology to be included in the microalgal biotechnology toolkit, but much additional research and development are necessary to further understand and validate the use of these tools, especially in relation to secondary metabolism.

## Adaptive laboratory evolution

Microorganisms have the ability to adapt rapidly to changing environments. Under severe stress, adaptation can occur via acquisition of beneficial phenotypes by random genomic mutations and subsequent positive selection [103, 104]. It is, therefore, not surprising that this plasticity of microorganisms has been harnessed for improving growth under stress conditions [21]. In contrast to the directed modification of particular enzymes and the rational engineering strategies, ALE enjoys the advantage of making the non-intuitive beneficial mutations take place within various regulatory regions and genes in parallel [105]. Moreover, engineering microalgae strains for overproduction of carotenoids or lipids usually requires the extensive genetic modifications, which often result in a great reduction of cellular fitness. On the contrary, ALE allows the phenotypic changes to be obviously connected with specific growth environment that results in the selection of the traits, which can not only overcome such negative effect but also lead to improvements in the physiological fitness of the strains. ALE has been widely utilized to change phenotypic and biological functions of many model organisms, such as *Escherichia coli* and *Saccharomyces cerevisiae* [105–107].

### Choice of stress conditions and equipment

During microbial ALE, a microorganism is cultivated under clearly defined conditions for prolonged periods of time, in the range of weeks to years, which allows the selection of improved phenotype. The selective stress serves as the first and also the foremost step for the success of ALE. It can be classified into two major categories, including the environmental stress and the nutrient stress [108]. In *Chlorococcum littorale*, ALE experiments were done using two different cycles: repeated long starvation (13 days of *N* = 0) lasting for 75 days and also repeated short starvation (6 days of *N* = 0) over a total period of 72 days. However, there was no difference shown in the production of biomass and lipid by the longer or shorter periods of starvation, suggesting that *C. littorale* might not fit the induction of the changes through ALE using nitrogen starvation as a stress factor [109]. If aiming to stimulate the growth performance of *C. littorale*, people should consider that ALE experiments are conducted under intensive selective pressure such as high light or high temperature. By contrast, ALE with nitrogen-starvation stress successfully improved the cell growth and lipid accumulation of *C. reinhardtii* [110], indicating the response to repeated stress is highly strain dependent. In this regard, one should pay attention to which stress condition can most potentially affect one’s working strain.

Another important parameter that affects the outcome of ALE experiment is the passage size, which decides how much of the population is permitted to propagate to each subsequent batch culture. If there is a beneficial mutation but is lost due to the population size bottlenecks, then the evaluation rate would be halted or slowed. It has been shown from the evolution experiments that the mutator hitchhiking can be seriously delayed among the smaller population bottlenecks [111]. However, enhancing the passage size would enhance the possibility of realizing the beneficial mutation, but also lead to increase in the resources needed to sustain. Due to this, the passage size is often determined by an individual’s schedule. For the microalgae, the impact of the passage size on the ALE experiment has not been systematic explored.

**Fig. 3:**
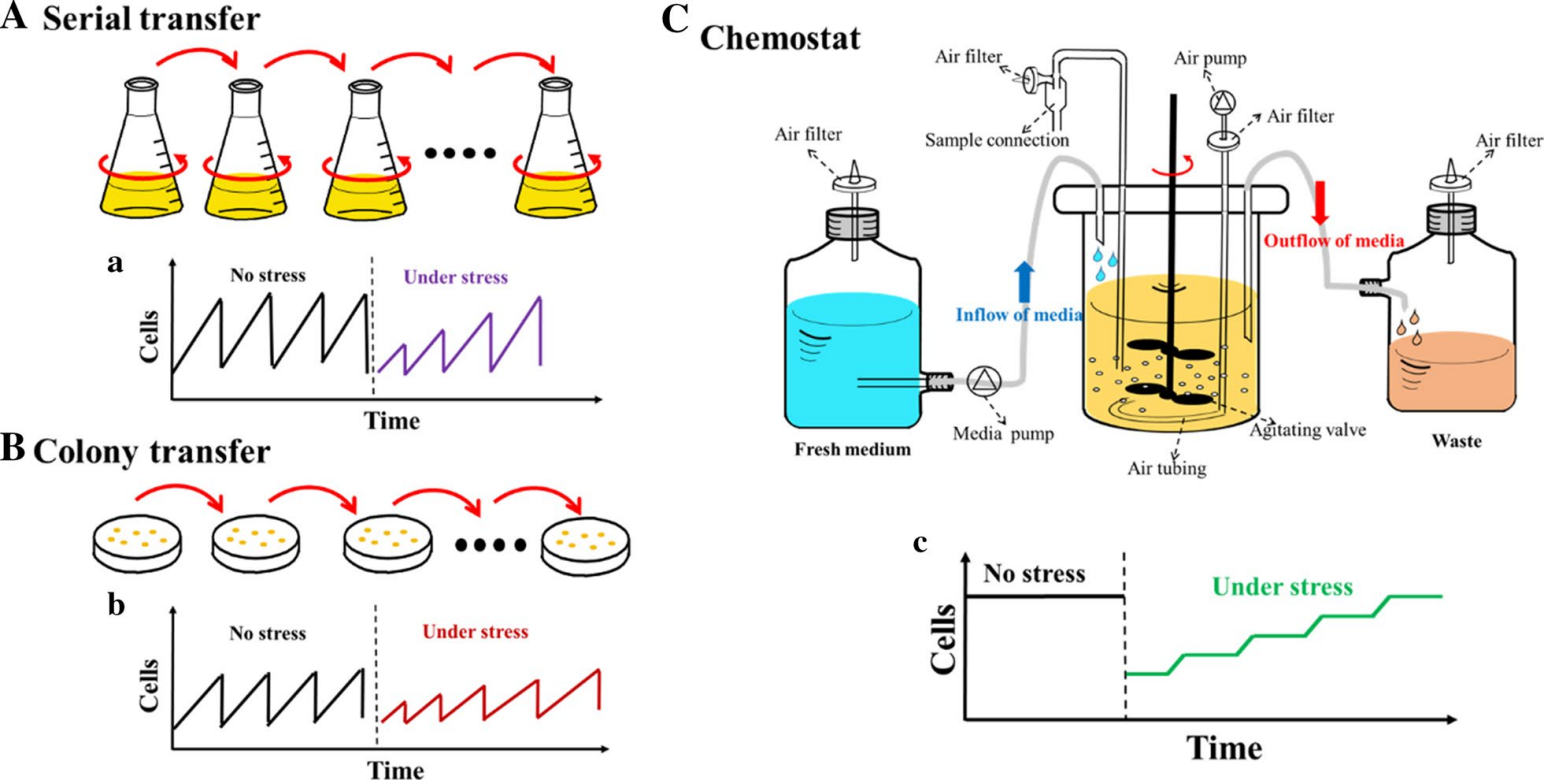
Adaptive laboratory evolution (ALE) can be performed in the laboratory using three broad approaches. **A** Serial transfer can be performed in shake flasks with liquid medium where nutrients will not be limited, and an aliquot of the culture is transferred to a new flask with fresh medium for an additional round of growth at regular intervals. **B** Colony transfer is similar to serial transfer, but is performed on plates with solid medium. **C** A chemostat comprises a culture vessel in which the population grows under continuous agitation and aeration. Fresh medium is added into the vessel at a defined rate and culture broth is harvested continuously during the process. The figures a, b, and c illustrate the number of cells that grew during ALE the processes shown in **A**, **B**, and **C**, respectively (This figure was modified from Jeong et al. [112])

In addition to the choice of selection pressure, ALE methods depend on culture equipment. In general, it is relatively easy to establish the ALE experiments. The common approaches usually include serial transfer, colony transfer or chemostat culture (Fig. 3). Serial transfer from a liquid medium and colony transfer from a solid medium were used for the sequential transfer of cells in repetitive cultures [112]. Put simply, an aliquot of the culture is transferred to a new flask or culture dish with fresh medium for an additional round of growth at regular intervals. Clearly, these easy setups have the advantage of cheap equipment and ease of massive parallel culture. By contrast, the central principle of the chemostat is that a stable equilibrium is achieved through the continuous addition of medium and simultaneous removal of culture broth [113]. In this constant steady state, defined nutrient- and environmental stresses can be finely tuned, and the growth rate of the cells can be experimentally controlled by modulating the rate of culture dilution. Although chemostat culture requires complex procedures that come with a higher cost, the larger cell population of the chemostat provides more genetic diversity than the smaller population of transfer techniques. Moreover, continuous selection using the chemostat technique can result in shorter times of ALE, which in turn can reduce the overall project cost.

### Development of stress-tolerant strains by adaptive laboratory evolution

In recent years, there were many ALE experiments being conducted successfully and applied to enhance the production of lipid or other high value-added products in microalgae (Table 3). Here, we summarized the most important workers regarding the ALE strategies with the aim of improving the stress tolerance of microalgae strains, as well as the production of lipid and carotenoids. The most common stress factors used for improving the performance of microalgae can also be divided into the categories of nutrient stress, environmental stress, oxidative stress, and natural selection stress.

**Table 3:** Typical adaptive laboratory evolution experiments with microalgae

Microalgal species	Selection pressure	Selection time	Performance	References
*Chlamydomonas reinhardtii*	Nitrogen limitation	50 days	The numbers of intracellular lipid bodies was massively increased	[110]
*Crypthecodinium cohnii*	Inhibitory concentration of glucose	650 days	High biomass and lipid accumulation was achieved	[115]
*Rhodococcus opacus*	High glycerol concentration	22 days	The conversion of glycerol into TAG was improved	[116]
*Rhodococcus opacus*	Phenol as sole carbon source	40 passages	The lipid production was increased by twofold	[117]
*Chlamydomonas reinhardtii*	Nitrogen starvation	84 days	Lipid productivity was increased by 2.36 times	[118]
*Chlorella vulgaris*	660 nm LEDs	114 days	Maximum biomass density was achieved	[119]
*Dunaliella salina*	Combined blue and red light	80 days	Increase of accumulation of carotenoids under combined blue and red light	[110]
*Phaeodactylum tricornutum*	Combined blue and red light	60 days	Increase of biomass production and fucoxanthin accumulation	[122]
*Chlorella* sp.	10% and 20% CO_2_	97 days	Enhanced CO_2_ fixation capability and carotenoids accumulation	[123]
*Schizochytrium* sp.	Agitation at 230 rpm	40 days	Maximum cell dry weight and DHA yield were observed	[131]

#### Nutrient stresses

The efficiency of nutrient utilization is an important aspect of microbial growth, and for industrial purposes, it may be governed by factors such as substrate costs or increased bioconversion rates. Although previous studies demonstrated that glucose is comparatively better compared with other carbon sources in stimulating DHA accumulation in *C. cohnii*, high glucose concentrations remain inhibiting cell growth and lipid accumulation [114]. To stimulate the high cell growth and release the substrate inhibition, ALE was performed for 650 days with gradually enhanced glucose concentration. Compared to the parent strain, glucose-tolerant *C. cohnii* strain yielded 15.49% more lipid accumulation [115]. The following metabolomics analysis suggested that the increased glucose tolerance was mediated by a positive regulation of glycerol, glutamic acid, succinic acid, and malonic acid, and negative regulation of tyrosine, lyxose, and fructose [115].

In addition to glucose, glycerol, the cheap by-product of biodiesel production, is also a potentially attractive substrate for the production of high value-added materials by fermentation. However, an engineered strain of *Rhodococcus opacus* MITXM-61 did not produce TAG on glycerol and grew poorly. Subsequently, an adaptive evolution strategy was applied by gradually adding glycerol into glucose/xylose medium, and the conversion of glycerol into TAG was improved successfully [116]. However, the genetic constitution of evolved strain was not unidentified. Surprisingly, Yoneda et al. [117] adaptively evolved *R. opacus* over 40 passages using phenol as sole carbon source. The endpoint strains showed higher phenol consumption rates and twofold higher lipid production from phenol than the wild-type strain. Whole-genome sequencing and comparative transcriptomics identified highly upregulated genes such as phenol monooxygenase reductase and shikimate transporter gene, implying that increased phenol import is more important than export in phenol tolerance in *R. opacus*. Moreover, lipid biosynthesis genes, including fatty acid synthesis gene (FAS) and desaturase genes, were also greatly up-regulated in evolved strain, which is consistent with the increase of lipid production.

In a somewhat different approach, *C. reinhardtii* was subjected to nitrogen limitation stress over 50 days to enhance the lipid production. After adaptive evolution, wild-type strain CC124 and starch-less mutant cells showed a 50% and 175% increase, respectively [110]. Furthermore, proteomics analyses showed that the key control protein [periplasmic l-amino acid oxidase (LAO1)] of carbon–nitrogen integration was specifically overexpressed. Moreover, the enzymes involved in lipid metabolism and lipid body-associated proteins (glutathione-S-transferases and esterase), were also induced during adaptive evolution [110]. When *C. reinhardtii* was conducted ALE combined with nitrogen-starvation stress for 84 days, the lipid productivity was increased by 2.36 times [118].

#### Environmental stresses

Among environmental stresses, adaptation of microalgae to unfavorable light conditions was investigated in the largest number of studies. The biomass productivity of microalgae is a key limiting factor of economic feasibility, especially in photosynthetic processes. A process spanning 114 days of ALE was used to improve the biomass productivity and cell density of *C. vulgaris* grown under less expensive lighting by 660 nm LEDs instead of the traditional but more expensive 680-nm LEDs [119]. In addition, ALE with light stress was also linked to increased carotenoids production. For example, Fu et al. [120] applied ALE to obtain mutants of *D. salina* (HI 001) with grown carotenoids accumulation, including the lutein and β-carotene under the combined red- and blue-light stress. Wild-type *D. salina* strains are not suitable for industrial production of lutein since they are sensitive to red light and unable to grow fast at high light intensities. By contrast, the ALE-derived strain *D. salina* (HI 001) can withstand high light stress, and therefore holds promise as an industrial lutein producer. Subsequently, the authors selected light quality, osmotic stress and nitrate concentration as three representative stressors to again enhance the lutein production in *D. salina* (HI 001) [121]. Similarly, in the marine diatom *Phaeodactylum tricornutum,* ALE with combined red- and blue-light stress also led to the increase of biomass production and fucoxanthin accumulation [122].

Moreover, to enhance the CO_2_ fixation ability of *Chlorella* sp., ALE was proposed under 10% CO_2_ condition to improve its CO_2_ tolerance, which led to increased accumulation of chlorophylls and carotenoids [123]. Nevertheless, this study did not investigate the physiological parameters and gene expression. Furthermore, a gradient-based adaptive evolution method was developed to increase the CO_2_ tolerance of *H. pluvialis* using 15% CO_2_ as selection stress. After 10 generations, the biomass and astaxanthin yields of the domesticated mutant in an atmosphere comprising 15% CO_2_ were 1.3 times and 6 times higher than in normal air, which might be attributed to the up-regulation expression of photosynthetic enzymes such as ATP synthase and RuBisCO genes [124].

In most of the microalgae, high-salt stress is beneficial to lipid accumulation, but itusually led to the decrease of photosynthetic pigments and oxidative damage. To obtain salt stress-tolerant microalgae, 1255 generations of ALE towards high-salt stress (200 mM NaCl) were performed in the green alga *C. reinhardtii*, and then yielded strains could grow as rapidly in high salt medium as the progenitor strain under normal conditions. Upon nitrogen depletion, the evolved cells were able to accumulate comparable amounts of lipid to those of the progenitor strain [125], which accompanied with significant up-regulation of calmodulin protein and down-regulation of glycerol/phospholipid acyltransferase and transporters. Similarly, this phenomenon was in agreement with an earlier study [126]. Recently, salt-resistant *Chlamydomonas* sp. strains were bred using a combined ALE and mutation strategy, which showed that the biomass production under high salinity was dramatically improved in the salt-resistant strains, but the lipid accumulation was decreased [127]. By contrast, when an ALE strategy combined with 30 g/L NaCl was applied in marine microalgae *Schizochytrium* sp., evolved strain exhibited a maximum biomass of 134.5 g/L and lipid yield of 80.14 g/L, resulting a 32.7% and 53.31% increase over the parental strain, respectively [128]. Despite of several benefits, the main limitation of ALE strategy is that most of the results are microalgae specific and its outcome may be different from strain to strain.

#### Oxidative stress

Apart from light, temperature and salt stress, oxidative stress also plays a vital role in cell proliferation and lipid accumulation in microalgae. Polyunsaturated fatty acids (PUFAs) imbue the cells with strong oxidation resistance [129, 130], and a high-oxygen stimulus might, therefore, induce the cells to produce more PUFA to protect themselves from oxidative injury. Based on this, ALE under continuous high oxygen was successfully applied to enhance the DHA production of *Schizochytrium* sp. [131]. It has been reported that microalgae with higher tolerance to oxidative stress are better suited for biofuel production [132]. In this regard, our recent study developed an innovative ALE strategy composed by cooperative two factors on the basis of the concomitant high salinity and low temperature to generate a stable improved *Schizochytrium* sp. strain that efficiently produce the lipid rich in PUFA. In this strategy, high salinity was used to trigger the lipid accumulation and promote the anti-oxidative defense systems of *Schizochytrium* sp., and low-temperature stress aimed to improve the PUFA content [133].

#### Natural selection stress

Compared to intentional adaptive evolution, natural adaptive evolution of strains possesses a greater probability for the culture to maintain the desired traits, although it is less likely to obtain a strain with desired characteristics. Using this strategy, Shin et al. [134] isolated and characterized a novel derivative of *C. reinhardtii*, which accumulated 116% and 66% more lipid under nitrogen- and sulfur-depleted conditions, respectively, than the ancestral strain. After evolution for 1880 generations in liquid medium under continuous light, the final *C. reinhardtii* strain had a 35% greater growth rate than the progenitor population, which was beneficial for lipid production [135].

Notably, the increasing application of ALE during recent years can be attributed to the ease of the access and decreasing costs of genome sequencing. Decreasing sequencing costs have led to increased investigation of “omics” technologies over the course of evolution, facilitating the study of fundamental stress-response mechanisms of microalgae. For example, an ALE approach was developed to evolve a strain of the microalga *Synechocystis* sp. with an improved growth performance under acid stress at pH 5.5. A subsequent whole-genome sequence analysis suggested that SNPs in certain genes are involved in acid stress tolerance [136]. Similarity, heat-tolerant strains of *Synechocystis* sp. were obtained by ALE, and the affected genes were identified by whole-genome sequencing [137]. Transcriptome analysis of salt-tolerant *C. reinhardtii* revealed gene expression differences between long-term and short-term acclimation [125]. Metabolomics and comparative transcriptomics have been used to elucidate the mechanism of butanol and phenol tolerance of evolved *Synechocystis* sp. and *R. opacus*, respectively [117, 138]. Genome-scale tools can identify the stress-responsive genetic elements responsible for an evolved phenotype. Furthermore, for a comprehensive and in-depth application of ALE strategy, genetic engineering and synthetic biology approaches should be developed to reintroduce point- or combined mutations into the parental strains in order to determine their specific phenotypic consequences.

## Challenges and future perspective

Microalgae have received a great amount of attention as potential cell factories for the production of bioactive compounds such as fatty acids and carotenoids. To overcome the negative effect of stress-based strategies, two-stage cultivation processes and the external addition of phytohormones or antioxidants can solve the conflicts between the accumulation of biomass and target product yields. However, the majority of these reports was conducted on the basis of laboratory-scale investigations and only were proven in minority microalgae species. Large-scale trials and economic feasibility studies of these strategies are still needed to verify their application. When moving from the lab production unit to a large scale, there are some relevant variables including large-scale photobioreactor, carbon dioxide availability, energy supply, as well as nutrient availability. Before, the behavior of photobioreactor has been discussed by Singh et al. [139], but they did not provide final direction. Therefore, above variables should be considered in the large-scale trials of these strategies.

ALE has become a proven powerful tool for the development robust stress-tolerant strains for improved production of lipid and carotenoids. However, there are no standardized procedures available for designing and performing ALE experiments. With the availability of computer simulations and automation, it is time to build new evolutionary model that simulates the dynamics of ALE experiment. Not only that, there are other problems hindering the development of ALE, such as the gene instability of evolved strain, longer operation time, and lower mutation rate evolution. To prevent degradation of strain performance, single colonies selected after ALE should be strictly performed. Periodical adaptive evolution is also a choice to strengthen gene stability of evolved strain. In the further, genetic engineering could be applied to regulate enzymes that involved in maintaining a high fidelity of DNA replication and repair, then the mutation rates in ALE process could be increased.

While a large number of studies focused on improving growth, only few studies were devoted to alleviate oxidative stress. Therefore, obtaining optimized microalgal strains with strong antioxidant potential is the key factor for stable and sustainable cultivation of microalgae, which is extremely important for industrial applications. In this regard, ALE could be applied to increase the oxidative stress tolerance of microalgae. Moreover, genetic engineering efforts need to be directed towards enhancing the antioxidant systems of microalgae. This can be done by overexpressing antioxidant enzymes and expressing stress tolerance genes from other organisms. In a recent study, overexpression of SOD in microalgae *Schizochytrium* sp. successfully alleviated oxidative stress and increased the lipid content [140]. It is anticipated that the dramatic progress of innovative technologies will offer more chances for unraveling the regulatory networks responsible for the cellular reactions to oxidative stress, which might offer guidance for the improved microbial lipid and carotenoids overproduction.

## Abbreviations

ABA: abscisic acid
ALE: adaptive laboratory evolution
APX: ascorbate peroxidase
CAT: catalase
DAH: diethyl aminoethyl hexanoate
DHA: docosahexaenoic acid
EPA: eicosapentaenoic acid
GSH: glutathione
IAA: indole-3-acetic acid
PUFA: polyunsaturated fatty acids
ROS: reactive oxygen species
SOD: superoxide dismutase
TAG: triacylglycerol
